# The Effects of Domestication on Secondary Metabolite Composition in Legumes

**DOI:** 10.3389/fgene.2020.581357

**Published:** 2020-09-18

**Authors:** Yee-Shan Ku, Carolina A. Contador, Ming-Sin Ng, Jeongjun Yu, Gyuhwa Chung, Hon-Ming Lam

**Affiliations:** ^1^Centre for Soybean Research of the State Key Laboratory of Agrobiotechnology and School of Life Sciences, The Chinese University of Hong Kong, Shatin, China; ^2^Department of Biotechnology, Chonnam National University, Yeosu, South Korea

**Keywords:** legume, domestication, secondary metabolite, defense, health benefit

## Abstract

Legumes are rich in secondary metabolites, such as polyphenols, alkaloids, and saponins, which are important defense compounds to protect the plant against herbivores and pathogens, and act as signaling molecules between the plant and its biotic environment. Legume-sourced secondary metabolites are well known for their potential benefits to human health as pharmaceuticals and nutraceuticals. During domestication, the color, smell, and taste of crop plants have been the focus of artificial selection by breeders. Since these agronomic traits are regulated by secondary metabolites, the basis behind the genomic evolution was the selection of the secondary metabolite composition. In this review, we will discuss the classification, occurrence, and health benefits of secondary metabolites in legumes. The differences in their profiles between wild legumes and their cultivated counterparts will be investigated to trace the possible effects of domestication on secondary metabolite compositions, and the advantages and drawbacks of such modifications. The changes in secondary metabolite contents will also be discussed at the genetic level to examine the genes responsible for determining the secondary metabolite composition that might have been lost due to domestication. Understanding these genes would enable breeding programs and metabolic engineering to produce legume varieties with favorable secondary metabolite profiles for facilitating adaptations to a changing climate, promoting beneficial interactions with biotic factors, and enhancing health-beneficial secondary metabolite contents for human consumption.

## Introduction

Climate change, farmland deterioration, and the resulting food insecurity are major challenges facing the world. An increase in food supply is required to feed the expanding human population. The cultivation of high-yield crops has been used as a strategy to improve food supply. Grain legumes have been suggested as the potential solution to maintaining food and protein security (Considine et al., 2017). Legumes are also beneficial for sustainable agriculture due to the reduced release of greenhouse gases compared to other crops (Stagnari et al., 2017). Besides the beneficial effects on the improvement of soil fertility, legumes could enhance the resistance of soil to ecosystem disturbance, possibly due to the enhanced soil food web complexity (Gao et al., 2020). In agriculture, legumes are common candidates for crop rotation for promoting the growth of other crops such as cereals (Bagayoko et al., 2000; Uzoh et al., 2019). In addition, legumes produce unique secondary metabolites such as isoflavones, which are beneficial to human health (Gepts et al., 2005; Ku et al., 2020). Legumes are known to protect humans from chronic diseases, including cardiovascular diseases, diabetes, obesity, osteoporosis, or even cancer (Kushi et al., 1999; Al-Anazi et al., 2011). Based on the mode of consumption, legumes can be classified into four groups: oil seeds, pulses, vegetable crops, and feed crops (McCrory et al., 2010). Examples of oil seeds are soybean and peanut (McCrory et al., 2010). Pulses are legumes, which are exclusively harvested as dry seeds, such as chickpea, lentils, and peas. Green bean and garden pea are examples of vegetable crops while clover and alfalfa are examples of feed crops (McCrory et al., 2010). Human selection of legumes during domestication has resulted in the alteration, and even loss of diversity, of secondary metabolite contents in these crops, directly and indirectly through the selection pressure on the genes that control the production of secondary metabolites. Understanding the differences in secondary metabolites, and the underlying genetic differences, between the domesticated legume cultivars and their wild progenitors would promote the preservation of legume accessions, which possess the genes for the biosynthesis of beneficial secondary metabolites. This knowledge will facilitate breeding programs and metabolite engineering to produce legume crops with favorable traits for adapting to the changing climate and for human pharmaceutical/nutraceutical use.

## Several Domestication-Related Traits Altered the Secondary Metabolite Contents

Domestication traits refer to morphological, biochemical, developmental, or physiological traits that are different between domesticated plants and their immediate wild progenitors (Abbo et al., 2014). A key part of domestication is the improvement of crop yield and harvestability compared to the wild progenitors (Dehaan et al., 2016). Several crop traits, including pod shattering, peduncle length, floral color, days to flowering, 100-seed weight, pod length, leaf length, leaf width, and seed number per pod, have been regarded as domestication-related traits (Lo et al., 2018).

Besides yield and harvestability related traits, other agronomic traits, such as seed size, appearance, and taste, are also subject to selection by breeders. These traits could be regarded as improvements due to post-domestication selection (Abbo et al., 2014). It has been suggested that the selection for larger seeds is related to facilitating single-seed planting (Kaplan, 1981). Breeders have also selected seeds of light colors. The ease of sowing and religious reasons have been proposed to be behind such conscious selections (Heiser, 1988). Therefore, seeds of modern legumes tend to have larger sizes and lighter colors compared to their wild counterparts. Moreover, the bitter taste of seeds has been intentionally eliminated through breeding (Muzquiz et al., 1994). Behind the loss of bitter taste is the loss of the corresponding bitter-tasting secondary metabolites such as alkaloids (Muzquiz et al., 1994).

During domestication, secondary metabolite compositions which facilitate cultivation and improve the appearance and taste of food grains were intentionally selected for by breeders. In some cases, the secondary metabolite composition may be unintentionally selected due to the close proximity of the genes or quantitative trait loci (QTLs) for secondary metabolite biosynthesis to those regulating other traits such as major nutrients and yield. The selection of favorable cultivation areas and the protection by breeders during crop growth limit natural selection pressures due to abiotic and biotic stresses. Domestication brings forth better yield, better taste, and better appearance but also reduces the availability of secondary metabolites in legumes. As a result, domesticated legumes are usually less resistant to biotic stresses compared to their wild counterparts (Muzquiz et al., 1994; Pavan et al., 2016; Bazghaleh et al., 2018; Abraham et al., 2019). The reduced availability of health-beneficial secondary metabolites (Muzquiz et al., 1994; Wang et al., 2010; Fernández-marín et al., 2014; Kaur et al., 2019) also limits the potential of legumes as sources of bioactive compounds for pharmaceutical use. For the growth of the legume plants, the loss of the secondary metabolites in modern cultivars possibly renders the plants more susceptible to abiotic stress and biotic stress. The importance of the secondary metabolites to combating these stresses will be introduced in section “The Roles of Secondary Metabolites in Combating Abiotic and Biotic Interactions.”

## Introduction to Secondary Metabolites in Legumes

### Definition of Plant Secondary Metabolites

Secondary metabolites are organic compounds derived from primary metabolism that serves key roles in defense and signaling in plants. They contribute to adaptive traits and ecological fitness, including defense mechanisms, tolerance to abiotic/biotic stresses, and interactions with insect pollinators, root-associated microbes, and herbivores. In contrast, primary metabolites are essential for cellular functions, such as growth, development, and reproduction. For example, secondary metabolites can attract insects for pollination or symbiotic rhizobia for nitrogen-fixing nodule formation. They can also be part of the defense mechanisms against herbivores, disease-causing bacteria, fungi, viruses, and parasites. There are also a wide range of secondary metabolites with pharmaceutical, nutraceutical, and toxicological values for humans (Wink, 2013). The contents of secondary metabolites vary among different plant species (Böttger et al., 2018). Legumes are rich in secondary metabolites, such as polyphenols, alkaloids, and saponins (Gupta, 1987).

### The Health Benefits of Secondary Metabolites From Legumes

In the recent past, many secondary metabolites in legumes were considered non-nutritive. For example, tannins, glycosides, alkaloids, and saponins affect the digestibility of beans (Gupta, 1987). However, more and more evidence suggests there are health benefits from the secondary metabolites of legumes (Dixon and Sumner, 2003). The health benefits of carotenoids, polyphenols, alkaloids, and saponins, all abundant in legumes, are discussed below and summarized in Table 1.

**Table 1:** Classification of secondary metabolites in legumes and their benefits to human health.

Groups	Sub-groups	Examples in legumes	Occurrence in legumes	Benefit(s) to human health	References
Polyphenols	Flavonoids	Quercetin, kaempferol	Widely distributed	Reduction in ischemic heart disease, reduction in body weight	(Knekt et al., 2002)
	Isoflavones	Genistein, daidzin	Soybean seeds	Phytoestrogen, antioxidant, antimicrobial and anti-inflammatory properties, reduction of risk in cardiovascular diseases, diabetes, obesity, and osteoporosis	(Křížová et al., 2019)
	Catechin	Catechin, epicatechin, gallo-catechin	Broad bean, chickpea, cowpea, kidney-bean, lentil, peanut	Reduction in heart disease, improvement of sperm motility and viability	(Arts et al., 2000; Hollman and Arts, 2000; Ojwang et al., 2013; Dias et al., 2016; López-cortez et al., 2016; Quintero-soto et al., 2018)
	Anthocyanins	Pelargonidin, cyanidin, malvidin, petunidin	Widely distributed	Antioxidant and anti-inflammatory properties, lipid peroxidation, DNA cleavage protection	(Acquaviva et al., 2003; Pietta et al., 2003; Rossi et al., 2012)
Terpenoids and steroid	Triterpenoid saponins	Saponins	Chickpea, soybean, lentils, peanut, common bean, and alfalfa sprouts	Reduction of cholesterol content, antimicrobial and anti-cancer properties	(Shi et al., 2004, 2014; Hassan et al., 2010; Man et al., 2010; Marrelli et al., 2016)
	Tetraterpenes	Carotenoids	Widely distributed	Antioxidant, better visual function, reduction of cardiovascular diseases	(Voutilainen et al., 2006; Roberts et al., 2009)
Alkaloids	Quinolizidine alkaloids (QA)	Sparteine	*Lupinus* spp.	Antimicrobial properties	(Romeo et al., 2018)
	Pyrroloindole alkaloids	Physostigmine	Ordeal bean	Treatment of Alzheimer’s disease and Parkinson’s disease	(Zhu et al., 2014; Kumar et al., 2015)
Peptides	Polypeptide	Lunasin	Soybean	anti-inflammatory properties, reduction of cholesterol content, antioxidant, anticancer and anti-atherosclerotic activities	(Jeong et al., 2002, 2003, 2007, 2009; Hsieh et al., 2017; Fernández-tomé and Hernández-ledesma, 2019)
	Protease inhibitors	Angiotensin-I converting enzyme inhibitors	Pea, chickpea, mung bean, soybean, lentil	Lowering blood pressure and risk of heart failure	(Zhang et al., 2018)
Amines	Polyamine	spermine, spermidine	Common bean, white clover, mung bean	Antioxidant activities, reduction of cardiovascular diseases	(Soda, 2010; Menéndez et al., 2019; Muñoz-Esparza et al., 2019)

#### Carotenoids

Carotenoids are a type of tetraterpenoids, ranging from bright yellow and orange to red, found in algae, photosynthetic bacteria, and plants, including carrot, pumpkin, tomato, sweet potato, and papaya. They can be classified into two groups, carotenes and xanthophylls (Roberts et al., 2009). Xanthophylls differ from carotenes by having oxygenated substituents in their molecules (Roberts et al., 2009). Carotenoids with unsubstituted β-rings, including α-carotene, β-carotene, and β-cryptoxanthin, act as provitamin A (Roberts et al., 2009). The carotenoid compositions in various legume seeds have been previously summarized (Tee et al., 1995). Lutein and zeaxanthin constitute the macular pigments in the retina of the mammalian eye. The oxygenated nature of the lutein and zeaxanthin molecules provides antioxidative protection for the eye from damage by free radicals (Roberts et al., 2009). The prevention of age-related macular degeneration by the consumption of carotenoid-rich foods has been recommended (Bernstein et al., 2016).

#### Polyphenols

Polyphenols are the major determinants of tissue colors, and generally possess antioxidative activities (Abbas et al., 2017). Polyphenols in plants can be classified into two groups: phenolic acids and flavonoids (Abbas et al., 2017). The occurrence and health benefits of phenolic acids in grain legumes have been previously summarized (Singh et al., 2017). Flavonoids are classified into several sub-classes: flavones, flavonols, flavanones, flavanonols, anthocyanins, flavanols, and isoflavones (Ku et al., 2020). Among flavonoids, isoflavones are only found in legumes. Flavonoids have multiple functions in plants, for example, mediating the responses to biotic and abiotic stresses, controlling the transport of auxins, acting as UV radiation-absorbing pigments to protect the plant against UV damage, attracting pollinating insects, interacting with rhizobia to initiate nodulation for symbiotic nitrogen fixation, and regulating defense against pathogens and herbivores through phytoalexin activities (Kumar and Pandey, 2013). For human health, it has been reported that flavonoids can act as protectants against cellular oxidation, inflammation, viral infections, and cancer (Kleemann et al., 2011). The molecular mechanisms of the health benefits of flavonoids have been recently reviewed (Ku et al., 2020).

#### Alkaloids

Alkaloids are nitrogen-containing organic heterocyclic compounds that are biologically active. Many alkaloids have pharmaceutical properties. For example, some alkaloids were found to have anti-malarial activities (Onguéné et al., 2013), anticancer activities (Gupta et al., 2015), and abilities to facilitate blood circulation in the brain and to prevent stroke (Kumar and Khanum, 2012). Moreover, several studies reported that alkaloids have potential therapeutic effects on neurodegenerative diseases, such as Alzheimer’s disease, Parkinson’s disease, and Huntington disease (Amirkia and Heinrich, 2014).

#### Saponins

Saponins are a group of terpenoids found in plants, including onion, ginger, garlic, ginseng, fenugreek, and legumes (Oakenfull, 1981; Sauvaire et al., 1996). These crops are important sources of saponins in the human diet (Oakenfull, 1981; Sauvaire et al., 1996). Chickpea, soybean, lentils, peanut, garden pea, broad bean, and alfalfa are rich in saponins (Oakenfull, 1981). The antibacterial and foaming properties of saponins led to the use of saponins as vaccine adjuvants (Marciani, 2018). In the human body, saponins can bind to bile salts to reduce cholesterol absorption (Marrelli et al., 2016). Moreover, in rats, it was shown that a saponin-rich diet resulted in the reduction of body weight, total cholesterol, triglycerides, very-low-density lipoproteins (VLDL), and low-density lipoproteins (LDL) in serum (Latha et al., 2011; Reddy et al., 2012). Alfalfa saponin extract (ASE) was found to have cholesterol-lowering effects (Wang et al., 2011; Marrelli et al., 2016). The treatment of rats with ASE led to the enhanced expression of cholesterol 7-alpha-hydroxylase (Cyp7a1), an enzyme involved in the bile acid biosynthetic pathway in the livers of hyperlipidemic rats (Marrelli et al., 2016). Besides, ASE treatment also enhanced the expression of low-density lipoprotein receptor (Ldlr), which promotes the uptake and clearance of LDL cholesterol in plasma (Marrelli et al., 2016). Moreover, saponins also have anti-microbial and antioxidant properties, and exhibit cancer-related immunomodulatory effects (Avato et al., 2006).

### The Roles of Secondary Metabolites in Combating Abiotic and Biotic Interactions

#### Polyphenols

Plant roots communicate actively with the soil microbes for mutualistic cycles. Flavonoids are important signaling molecules for the legume-microbe interactions. The ability to form nitrogen fixing nodules with rhizobia is a unique characteristic of legumes (Hirsch et al., 2001). Such mutualism between legume and rhizobium is initiated by flavonoids. Flavonoids released from roots attract rhizobia to migrate toward the roots and stimulate the *nod* genes, which are essential genes to synthesize Nod factors for infecting the plants (Spaink, 1995). Flavonoids in the root exudates of various legumes for attracting rhizobia have been summarized in a previous review (Haldar and Sengupta, 2015). Moreover, flavonoids stimulate the germination of mycorrhizal fungus spores and enhance hyphal growth (Abdel-lateif et al., 2012). Mycorrhizal fungi form hyphae which penetrate plant roots for the transport of nutrients in rhizosphere to the host plant (Harrison, 2005).

The importance of polyphenols to combating abiotic stress has been discussed in recent reviews (Di Ferdinando et al., 2014; Isah, 2019; Sharma et al., 2019). The antioxidating characteristics of polyphenols help alleviate the oxidative stress brought forth by abiotic stress (Di Ferdinando et al., 2014; Isah, 2019; Sharma et al., 2019). A recent method for screening legume crops for abiotic stress tolerance suggested the accumulation of anthocyanin, which is also an osmolyte, as one of the indicators of abiotic stress tolerance of legume crops (Sinha et al., 2020).

#### Strigolactones

Based on the molecular structure, strigolactones belong to a group of lactone, which is derived from carotenoid (Jia et al., 2018). Functionally, strigolactones are plant hormones that are released by roots to attract symbiotic arbuscular mycorrhizal fungi and induce the germination of parasitic weed seeds (Jia et al., 2018). Strigolactones have been identified from a broad range of legumes, including *Arachis hypogaea*, *Astragalus sinicus*, *Cicer arietinum*, *Glycine max*, *Lupinus albus*, *Medicago sativa*, *Phaseolus vulgaris*, *Pisum sativum*, *Psophocarpus tetragonolobus*, *Trifolium incarnatum*, *Vicia faba*, and *Vigna angularis* (Yoneyama et al., 2008).

A study showed that the expression of several secretory proteins of *Rhizophagus irregularis*, an arbuscular mycorrhizal fungus, was induced by strigolactone treatment (Tsuzuki et al., 2016). Among these proteins, Strigolactone-Induced Putative Secreted Protein 1 (SIS1) showed the highest induction fold by both strigolactone treatment and *Medicago truncatula* root symbiosis. SIS1 is important for colonization and the formation of stunted arbuscules (Tsuzuki et al., 2016). Therefore, the strigolactone-induced is an essential protein for the symbiosis (Tsuzuki et al., 2016).

Broomrapes, especially *Orobanche crenata*, are believed to be the major parasitic weeds of legumes. The effects of the parasitic weeds on legumes include local damage of the plants and yield loss (Rubiales and Fernández-Aparicio, 2012). The germination of *Orobanche* seeds is induced by strigolatones (Yoneyama et al., 2008).

#### Alkaloids and Saponins

Alkaloids and saponins are known for contributing to the bitter taste of plants (Drewnowski and Gomez-Carneros, 2000). The toxicity of alkaloids has been reported (Wink, 2013). Several studies report that alkaloids and saponins are related to the resistance to herbivores. For example, yellow lupin cultivar with higher level of alkaloids in the leaves is more resistant to aphid than the cultivar with lower level of alkaloids (Adhikari et al., 2012). The removal of the bitter taste from modern lupin cultivars has enabled them to be a protein source in animal feed to reduce the dependence on soybean (Abraham et al., 2019). However, “sweet” lupins are more susceptible to predators (Muzquiz et al., 1994). Saponins have been thought to be responsible for the resistance to insect attacks, as the saponin preparation garden pea (*Pisum sativum* L.) resistant to Azuki bean beetle (*Callosobruchus chinensis* L.) inhibited the development of the beetle (Applebaum et al., 1969).

## Alterations of Secondary Metabolite Profiles in Legumes During Domestication

### Polyphenols and Carotenoids Determine the Colors of Seeds and Flowers

The seed coat color is mainly determined by polyphenols such as tannins (Heiser, 1988; Espinosa-Alonso et al., 2006). It is common for the pigmentation patterns of domesticated crops to be altered compared to their wild relatives. The loss of pigment in the seed coat of cultivated *P. vulgaris* is an obvious example of the effects of domestication (McClean et al., 2018). In a survey of 18 *Lablab purpureus* (*L. purpureus*) germplasms, including wild, semi-domesticated, and cultivated accessions, it was found that all the wild accessions have gray-brown and mottled seed coat (Maass, 2006). However, cultivated accessions display a spectrum of seed coat colors, including cream-white, cream, tan, and black (Maass, 2006). Unlike the wild accessions, some cultivated accessions do not have mottled seed coats (Maass, 2006). Among 11 landraces and two cultivated accessions of peanut (*Arachis hypogaea* L.), it was found that all the cultivated accessions have a single seed coat color: tan (Husain and Mallikarjuna, 2012), while the landraces are either red or tan (Husain and Mallikarjuna, 2012). Some landraces even have variegated seed coats (Husain and Mallikarjuna, 2012). In another study, it was shown that cultivated peanut (*A. hypogaea*) could have purple, brown, red, or white seed coats and some have variegated seed coats (Bertioli et al., 2011). In a survey of a soybean population consisting of 1,957 domesticated and 1,079 wild accessions, it was found that almost all wild accessions have purple flowers and black seed coats (Jeong et al., 2019), whereas the domesticated soybean accessions have more diverse seed coat colors, including colorless (yellow or green seeds), brown, or black, and more diverse floral colors, including white or purple (Jeong et al., 2019). In another study on 110 cultivated, 130 landrace, and 62 wild soybean accessions, it was reported that all cultivated accessions have yellow seeds, and landrace accessions have yellow, green, brown, or black seeds, while all the wild accessions have black seeds (Wang et al., 2018). Similarly, the modern cultivated pea cv. Cameor (*P. sativum*) has transparent seed coat while the wild accession (*P. sativum* subsp. *elatius* JI64) has pigmented seed coat (Smýkal et al., 2014). In another study on cultivated (*Lens culinaris* ssp. *Culinaris*) and wild lentils (*Lens culinaris* ssp. *orientalis*, *L. culinaris* ssp. *odemensis*, *L. culinaris* ssp. *tomentosus*, *Lens nigricans*, and *Lens ervoides*, *Lens lamottei*), although wild accessions do not necessarily have darker seed coats, wild accessions have more complexed patterns on the seed coats (Singh et al., 2014). The seed coats of the cultivated accessions have either no or dotted patterns (Singh et al., 2014). However, many of the wild accessions have marbled pattern on seed coats (Singh et al., 2014). For chickpea (*C. arietinum*), the light color of the cultivated seeds is thought to be non-existing in wild accessions (Penmetsa et al., 2016). The seed coat color is related to the defense against herbivore. It has been suggested that a black seed coat protects the seed from night-time foragers (Porter, 2013).

Polyphenols also give rise to the colors of flowers (Wiesner et al., 2017). In cowpea, cultivated accessions have a wide range of floral colors while most of the wild accessions have only purple flowers (Lo et al., 2018). Similarly, cultivated soybean accessions have purple, white, or other colors of flowers (Sundaramoorthy et al., 2015; Jeong et al., 2019), whereas most of the wild soybean accessions have only purple flowers (Sundaramoorthy et al., 2015). On the contrary, in common bean, most of the cultivated accessions have only white flowers while the wild accessions have white, pink, or purple flowers (García et al., 1997). Cultivated lentils (*L. culinaris* ssp. *Culinaris*) have white or purple flowers but some wild lentils, *L. culinaris* ssp. *odemensis*, *L. culinaris* ssp. *tomentosus* and *Lentil ervoides*, have only purple flowers (Singh et al., 2014). The white flowers of cultivated chickpea (*C. arietinum*) is thought to be non-existing in wild accessions (Penmetsa et al., 2016). For pea, cultivated peas (*P. sativum*) usually have white flowers while purple flowers are found in wild peas (Hellens et al., 2010). The contrasting flower colors contributed to the establishment of the Mendel’s Laws.

#### The Co-Evolution of Seed and Floral Colors With Foragers and Pollinators

As discussed above, cultivated legumes usually have lighter seed coat colors compared to the wild counterparts. During domestication, light seed coat colors have been preferred by farmers. The loss of color is associated with the loss of secondary metabolites, such as tannins (Heiser, 1988). As mentioned before, a dark seed coat may protect the seeds from night-time foragers in the wild (Porter, 2013). However, the potential increase in loss of sown seeds to wild animals may not be significant as farmers usually have measures to keep foragers away from crops. Another example is the loss of bitter compounds, such as alkaloids and saponins in domesticated legumes. The loss of such compounds would have enhanced the loss of seeds due to foraging by animals and is usually not advantageous for the survival of the crops without the protection provided by farmers. Therefore, the loss of bitter compounds in domesticated legumes is also known as a conscious selection by breeders during domestication. In soybean, most of the elite cultivated soybean seeds are yellow. It was found that the stay-green *G* gene is associated with green seeds and it controls seed dormancy, but is lost in elite cultivated soybean seeds (Wang et al., 2018). In the survey of 110 cultivated, 130 landrace, and 62 wild soybean accessions, it was found that the *G* genotype is present in only 4% of the cultivated accessions, 21% of the landraces while it is found in 100% of the wild accessions (Wang et al., 2018).

It has been suggested that floral color has co-evolved with pollinators such as birds and bees. Bees tend to be attracted to yellow flowers while birds tend to prefer red flowers due to their different visual sensitivities (Toon et al., 2014). Bee-pollinated plants usually have yellow, white, or blue flowers while bird-pollinated plants usually have red flowers (Toon et al., 2014). The transition from bee-pollination to bird-pollination of Australian egg-and-bacon pea is related to the number of bird species in the geographical region where the plants grow (Toon et al., 2014). The yellow color of the *Lotus* flower, together with the orientation, size, petal morphology, sucrose-dominant nectar composition, and scent of the flower, was reported as a factor contributing to the transition to pollination by birds (Cronk and Ojeda, 2008).

#### Carotenoid Level Is Related to Seed Dispersal by Animals

Besides polyphenols, carotenoids also play a role in determining tissue colors. During domestication, the profitability of seeds is a major concern for farmers. Therefore, genotypes with reduced seed dispersal, including through pod shattering and seed dispersal by animals, were actively selected for by breeders and farmers. In a study on the seeds of 10 legume genera: *Arachis* (peanut), *Cicer* (chickpea), *Glycine* (soybean), *Lathyrus* (vetch), *Lens* (lentil), *Lupinus* (lupin), *Phaseolus* (bean), *Pisum* (pea), *Vicia* (fava bean), and *Vigna* (cowpea), drastic changes in the levels and compositions of carotenoids in seeds were found in domesticated cultivars compared to their wild counterparts (Fernández-marín et al., 2014). An average of 48% reduction in carotenoids was found in the seeds of these 10 legumes. Besides, the compositions of carotenoids were more complex in the wild species of *Cicer*, *Glycine*, *Lathyrus*, *Lens*, *Lupinus*, and *Vigna*. In the study, neoxanthin, violaxanthin, lutein epoxide, and antheraxanthin were only found in the wild species but not the domesticated varieties. It was suggested that seeds with lower carotenoid levels are less attractive to seed dispersers (Fernández-marín et al., 2014). In contrast, attracting seed dispersers has been suggested to be an adaptation of wild legumes (Brǿnnvik and von Wettberg, 2019). It was suggested that seed dispersal by birds is an important factor contributing to the widespread of *P. vulgaris* from Mexico to South America (Brǿnnvik and von Wettberg, 2019).

### Isoflavones Are Unique to Legumes

Isoflavones are a sub-class of flavonoid uniquely found in legumes. Soybean is a rich and common source of isoflavones for human consumption (Ku et al., 2020). In a study of seed isoflavone contents using 209 wild, 580 landrace, and 106 cultivated soybean accessions, it was found that landraces had the highest average level of total seed isoflavone, followed by wild accessions and then cultivated accessions (Wang et al., 2010). The higher average total seed isoflavone content in landraces compared to cultivated accessions was also reported in another study using 927 landraces and 241 cultivars (Azam et al., 2020). For individual isoflavone contents, it was suggested that high genistin and glycitin contents, with low daidzin levels, were artificially selected for. The significantly lower daidzin contents lead to the lower average total seed isoflavone levels in cultivated accessions compared to wild accessions (Wang et al., 2010). There are debates over the reasons behind the artificial selection of such seed isoflavone traits in domesticated legumes. Regarding seed nutrient content, a negative correlation between the total isoflavone level and the protein level has been reported in seeds (Primomo et al., 2005; Morrison et al., 2008; Liang et al., 2010; Smallwood et al., 2014), and a positive correlation between total seed isoflavone level and seed oil level has also been reported (Morrison et al., 2008; Liang et al., 2010). However, there has also been a report on the negative correlation between total seed isoflavone level and seed oil level (Smallwood et al., 2014). The total seed isoflavone has also been correlated to yield (Primomo et al., 2005; Smallwood et al., 2014; Zhang et al., 2014), as well as the resistance against pathogens (Carter et al., 2018). When two soybean cultivars, RCAT1004 and DH4202, which are resistant and sensitive to cyst nematodes respectively, were grown in a cyst nematode-infested environment, the resistant cultivar had a higher seed isoflavone level (Carter et al., 2018). A putative QTL related to cyst nematode susceptibility was found close to that related to total seed isoflavone content (Carter et al., 2018). During domestication, besides the deliberate selection for reduced seed isoflavone level to reduce the bitterness of the seed, the isoflavone level may also be unintentionally selected together with other desirable traits, such as nutrient composition, yield, and resistance to biotic stress.

### Alkaloid and Saponin Contents Are Related to Taste-Focused Breeding

The bitter taste of legume seeds tends to be eliminated during domestication. For example, domesticated lupin cultivars are less bitter than the wild relatives, which have significantly higher levels of alkaloids in their seeds. Modern lupin cultivars are referred to as “sweet” lupins. In a survey of 20 sweet lupins and 29 bitter lupins, the bitter taste of lupins was found to be positively correlated to the seed alkaloid content, with lupanine being the main alkaloid (Muzquiz et al., 1994). Although seed saponin level has been correlated to the bitterness of seeds in general (Mohan et al., 2016), it may not be related to the bitterness of lupin seeds. In a survey of the seed saponin contents in sweet vs. bitter lupins, the level of saponin was undetectable in the seeds of both sweet and bitter varieties of *L. albus* (Shim et al., 2003).

Saponin is also a contributing factor to the bitterness of seeds (Okubo et al., 1992). It was found that many of the wild ancestors of *Vigna* spp. are more resistant than their cultivated counterparts to *Callosobruchus chinensis* or *Callosobruchus maculatus* (Tomooka et al., 2000). It is possible that the drop in saponin contents when legumes became domesticated is related to the loss of insect resistance capability in cultivated species. Several wild chickpea accessions have higher seed saponin levels than cultivated chickpea accessions (Kaur et al., 2019). Several wild pigeonpea (*Cajanus scarabaeoides*) accessions have higher seed saponin contents than the cultivated pigeonpea accessions (*Cajanus cajan*; Sekhon et al., 2017). However, cultivated pigeonpea accessions do not necessarily have lower seed saponin contents than the wild accessions (Sekhon et al., 2017). Seed saponin content is not the sole factor leading to the insect resistance of legumes.

Besides total seed saponin content, individual saponin components in legumes are also studied. Saponins can be classified into four groups: group A saponins, group B saponins, group E saponins, and 2,3-dihydro-2,5-dihydroxy-6-methyl-4H-pyran-4-one (DDMP) saponins (Sawai and Saito, 2011; Krishnamurthy et al., 2013). The aglycone form of group A saponins is named soyasapogenol A, while that of DDMP saponins is named soyasapogenol B. The basic structure of soyasapogenol A and B is β-amyrin. Soyasapogenol A is a β-amyrin with a hydroxyl group at C-21, C-22, and C-24, while soyasapogenol B has a hydroxyl group at C-22 and C-24 only (Sawai and Saito, 2011). DDMP saponins are relatively unstable and are often degraded into group B and group E saponins during food processing (Sundaramoorthy et al., 2019). Among the various groups of saponins, group A saponins, which have an acetylated oligosaccharide chain attached to C-22 of soyasapogenol A, are thought to be mostly responsible for the undesirable taste of soybean seeds (Shiraiwa et al., 1991).

In a survey of saponin compositions among 800 cultivated soybean accessions and 329 wild soybean accessions, it was found that the saponin type Aa was predominant in cultivated soybean accessions, while the saponin type AaBc was predominant in wild soybean accessions (Tsukamoto et al., 1993). In another survey of the total seed saponin levels in 17 wild and one cultivated legumes, it was found that the total saponin level was highest in *Glycine soja* (*G. soja*; wild soybean; Shim et al., 2003). In a study of seed saponin composition of 3,025 *G. soja* accessions, diverse compositions of seed saponins were found among the accessions (Krishnamurthy et al., 2013). Moreover, naturally occurring wild soybean mutants that lack group A saponins were found (Krishnamurthy et al., 2013; Takahashi et al., 2016; Rehman et al., 2018). Wild legumes do not necessarily have higher seed saponin contents. Instead, the diverse genetic backgrounds among wild legumes allow the discovery of novel allelic forms for desirable seed saponin compositions.

### Polyphenols and Strigolactones Are Related to Biotic Interactions

Flavonoids are signaling molecules for legume-microbe interaction (Abdel-lateif et al., 2012). In a test of nodulating capability of *Rhizobium japonicum* (*R. japonicum*), it was found that all the strains of *R. japonicum* in the test could nodulate cowpea, sirato, and wild soybean (Heront and Pueppket, 1984). However, nine out of the 11 strains could not form infection threads with two of the three commercial soybean cultivars in the test (Heront and Pueppket, 1984). In another study, after inoculating 36 *G. soja* (wild soybean) accessions with *R. japonicum*, 20 formed normal nodules while 16 could not form nodules or formed abnormal nodule-like structures (Pueppke et al., 1998). It was hypothesized that the different nodulating phenotypes were due to the different flavonoid profiles in the root exudates (Pueppke et al., 1998). However, the flavonoid profiles of root exudates are similar between the nodulating group and the non-nodulating group (Pueppke et al., 1998). The flavonoid profiles of root exudates were also compared between wild soybean accessions and the cultivated soybean Peking (Pueppke et al., 1998). Although many of the wild soybeans showed a more complexed root exudate profile, a strong correlation between the different root exudates and the nodulating phenotypes was not found (Pueppke et al., 1998). The effects of domestication on the flavonoid profiles in legume root exudates remain unclear. On the other hand, the root polyphenol compositions of wild lentil (*Lens ervoides*) and cultivated lentil (*Lens culinaris*) were compared after the infection of *Aphanomyces euteiches*, which is a legume pathogen (Bazghaleh et al., 2018). The wild lentil was more tolerant to *A. euteiches* than the cultivated lentil pathogen (Bazghaleh et al., 2018). The wild lentil generally had higher levels of polyphenols compared to the cultivated lentil (Bazghaleh et al., 2018). Although the amount of legume species and accessions is not enough to conclude the effect of domestication on the root polyphenol compositions after pathogen infection, genotypic difference exists between wild and cultivated legumes and is associated with polyphenol accumulation in roots under biotic stress.

Strigolactones are stimulants of seed germination (Brun et al., 2018). The yield of faba bean (*V. faba*) is also limited by parasitic weeds. Faba bean (*V. faba*) germplasms resistant to parasitic weeds, broomrape (*Orobanche* and *Phelipanche* spp.) were found (Fernández-Aparicio et al., 2014). The resistant germplasms have low or undetectable levels of strigolactones in the root exudates at all plant ages (Fernández-Aparicio et al., 2014). It was suggested that the screening of germplasms with low strigalactone levels in root exudates is a strategy to breed for weed resistant germplasms. Like faba bean (*V. faba*), most of the commercial pea (*Pisum sativum* L.) cultivars are susceptible to the attack by crenate broompape (*O. crenata* Forsk.), which is a parasitic weed of legumes (Pavan et al., 2016). In a screen of *O. crenata* resistant pea germplasms, a landrace pea germplasm was selected. Repeated self-pollination of the landrace germplasm resulted in the *O. crenata* resistant line ROR12 (Pavan et al., 2016), which exhibited several unique characters: (1) compared to a *O. crenata* susceptible cultivar, the root exudates of ROR12, which had a lower strigolactone level, had a lower capability to stimulate the germination of *O. crenata* seeds; (2) in the field, the number of *O. crenata* shoots per host plant of ROR12 was lower; and (3) the emergence of *O. crenata* on ROR12 was delayed. It was proposed that the resistance to *O. crenata* was related to the reduced strigolactone level in the root exudates (Pavan et al., 2016).

Strigolactones are also involved in legume-microbe interaction. The treatment of synthetic strigolactone (GR24) to pea (*Pisum sativum* L.) roots enhanced the nodule number on the roots due to *Rhizobium leguminosarum* bv. *viciae* (RLV248) inoculation (Foo and Davies, 2011). Mutant *rms1* of pea (*Pisum sativum* L.) had undetectable levels of orobanchol and orobanchyl acetate and a low level of fabacyl acetate in the root exudates (Foo and Davies, 2011). Compared to the wild type, *rms1* mutant had less nodules on the roots after being inoculated with *R. leguminosarum* bv. *viciae* (RLV248; Foo and Davies, 2011). Commercial legume germplasms usually have lower levels of strigolactones in the root exudate (Fernández-Aparicio et al., 2014; Pavan et al., 2016).

The low levels of strigolactones may result in the reduced number of nodules on the roots. However, the nodulating phenotype may not be of consideration during domestication as the application of nitrogen fertilizer is a common practice during domestication.

## Genes That Regulate the Different Secondary Metabolite-Related Traits

Several methods are currently being employed to identify the genes and mutations underlying legume domestication phenotypes (Olsen and Wendel, 2013). In general, secondary metabolites are present at higher levels in wild progenitors than in the domesticated counterparts as a result of artificial selection (Nagl et al., 1997; Lindig-Cisneros et al., 2002; Gepts, 2014). The selection of cultivars based on ease of farming and other commercial attributes may have occurred at the expense of potentially beneficial secondary metabolites. The reduction in genetic diversity is one of the main impacts of domestication. However, the genetic richness of wild populations can be used to improve cultivated legumes. Traditional plant breeding is a millenary process for the improvement and development of new crop varieties. According to breeding objectives, new legume varieties are produced by crossing parents with desired traits and selecting among segregating progenies those individuals with both high yield and the target trait. In this way, pest-resistant varieties have been developed with genetic resistance to pathogens (Lavaud et al., 2015). Traits related to pigmentation and defense against pathogens or herbivores are characteristically domestication-related traits governed by secondary metabolites. Besides biosynthesis-related genes, transport-related genes are also important. The roles of transporters, including ATP-binding cassette (ABC) transporters and multidrug and toxic compound extrusion (MATE) transporters in secondary metabolite secretion and accumulation have been summarized in previous reviews (Yazaki, 2005; Ku et al., 2020). In this section, examples of genes and loci controlling secondary metabolite biosynthesis and transport in legumes will be discussed.

### Biosynthesis-Related Genes

#### Pigmentation-Related Traits

**Figure 1:**
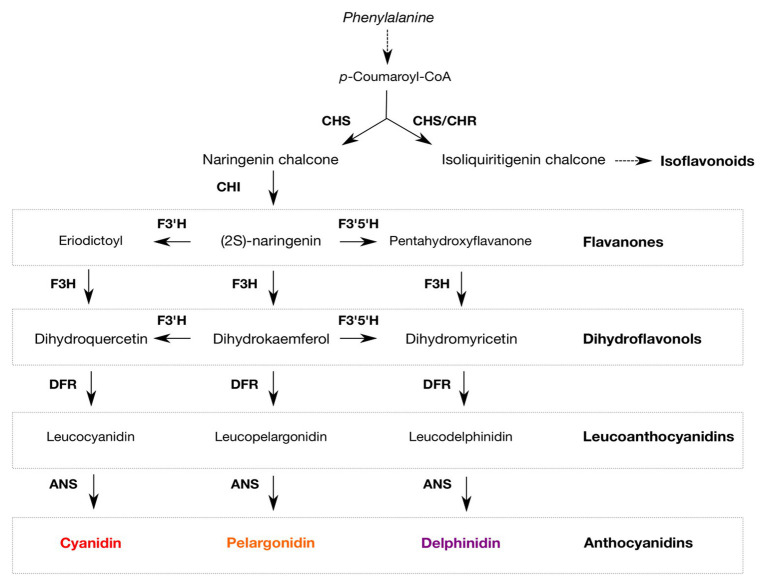
Schematic representation of the flavonoid biosynthetic pathway. Enzymes involved in the pathway are indicated in bold: chalcone synthase (CHS), chalcone reductase (CHR), flavone 3-hydroxylase (F3H), flavonoid 3'-hydroxylase (F3'H), flavonoid 3',5'-hydroxylase (F3'5'H), dihydroflavonol 4-reductase (DFR), and anthocyanidin synthase (ANS).

Polyphenols are the major determinants of tissue colors, including the colors of seed coats of legumes, both by their presence and their quantities (Espinosa-Alonso et al., 2006). The major polyphenols responsible for seed coat color in legumes are flavonoids, such as anthocyanins, flavonol glycosides, and proanthocyanidins (condensed tannins). Flavonoid quantities vary according to the seed developmental stages, genotypes, and species. The biosynthetic pathway leading to the biosynthesis of flavonoids has been elucidated and is conserved among seed-producing plants. Flavonoids and isoflavonoids are derived from the phenylpropanoid pathway (Dastmalchi and Dhaubhadel, 2014). Many genes in this pathway, including enzymes, transporters, and regulatory factors, have been characterized. The first committed step is the formation of a bicyclic tetrahydroxy chalcone (naringenin chalcone) catalyzed by a chalcone synthase (CHS). Legumes produce an additional trihydroxy chalcone (THC), isoliquiritigenin chalcone (Dastmalchi and Dhaubhadel, 2014). This THC is the end product of the coupled activities of CHS and the legume-specific chalcone reductase (CHR). Compounds such as daidzein, medicarpin, and glyceollin are derived from isoliquiritigenin. Flavonoid production follows the conversion of naringenin chalcone to (2S)-naringenin by chalcone isomerase (CHI). Flavone 3-hydroxylase (F3H) catalyzes the hydroxylation of (2S)-naringenin, eryodictyol, and pentahydroxyl flavanones to yield (2R,3R)-dihydrokaempferol, dihydroquercetin, and dihydromyricetin, respectively (Tanaka et al., 2008). Flavonoid 3'-hydroxylase (F3'H) and flavonoid 3',5'-hydroxylase (F3'5'H) catalyze the hydroxylation of flavanones, flavanols, and flavones, and determine the structures of flavonoids and anthocyanins (Tanaka, 2006). Other enzymes in the pathway include dihydroflavonol 4-reductase (DFR) and anthocyanidin synthase (ANS). The biosynthesis pathway of flavonoids is illustrated in Figure 1.

Pigmentation mechanisms have been studied in different legumes. A transcriptomic analysis was performed to identify the genes associated with seed coat color in peanut (*A. hypogaea*; Wan et al., 2016). Lower proanthocyanidin and anthocyanin contents were detected in a peanut mutant with a brown cracking seed coat (*pscb*). Transcriptomic analyses revealed that the structural genes of the phenylpropanoid biosynthetic pathway were downregulated in the *pscb* mutant, while the genes related to melanin production were upregulated at the late developmental stages. This expression pattern was consistent with the higher melanin content in the *pscb* mutant compared to the wild type. Differential expression analyses of RNA-seq data between the wild type and *pscb* mutant revealed three candidate genes (*c36498_g1*, *c40902_g2*, and *c33560_g1*) as being responsible for the seed coat color trait. *C33560_g1* encodes a R2R3-MYB transcription factor. Its homologs in *Arabidopsis* and apple are associated with the regulation of the phenylpropanoid biosynthesis pathway (Rowan et al., 2009; Vimolmangkang et al., 2013). *C36498_g1* and *c40902_g2* encode a caffeoyl-CoA O-methyltransferase and a kinesin-4-like protein, respectively. Putative functions of the encoded proteins were associated with cell wall organization.

Soybeans cultivated for the commercial market are either completely yellow or have pigmentation restricted to the hilum (Palmer et al., 2004). Wild soybeans accumulate flavonoids and anthocyanins within the entire epidermal layer of the seed coat, giving them a black or brown color (Todd and Vodkin, 1993; Song et al., 2016). Quantitative trait loci (QTL) governing seed coat color in soybean have been identified using genetic and genomic analyses to elucidate the genetic changes that resulted in this domestication trait (Todd and Vodkin, 1996; Tuteja et al., 2004, 2009; Song et al., 2016). The *I*, *R*, and *T* loci were found to be involved in the flavonoid biosynthesis pathway (Palmer et al., 2004; Yang et al., 2010). The *I* locus on chromosome eight inhibits pigmentation of the seed coat. There are four alleles (*I*, *i^i^*, *i^k^*, and *i*) at the *I* locus where *I* and *i^i^* are the two dominant forms (Song et al., 2016). The presence of the *I* allele results in the absence of pigmentation and a yellow seed coat at maturity. This allele contains an inverted repeat of the *CHS* gene cluster. This structure triggers posttranscriptional gene silencing (PTGS), which inhibits the expression of *CHS* gene family members and their functions in the flavonoid biosynthesis pathway (Tuteja et al., 2004). The *i^i^* allele inhibits pigmentation, resulting in a yellow seed coat with a pigmented hilum (Palmer et al., 2004). Meanwhile, the recessive *i^k^* and *i* alleles allow pigment production, with the *i^k^* allele restringing pigments to the saddle and hilum regions of the seed coat (Palmer et al., 2004). The *R* and *T* loci determine the type and accumulation of pigments in the seed coat (Buzzetl et al., 1987; Todd and Vodkin, 1993). Higher flavonoid and anthocyanin contents of seeds are currently of great interest due to the antioxidant properties and flavors of these compounds. Recently, the wild soybean reference genome of *G. soja* W05 was used to identify additional alleles of the causal structural gene variation that controls soybean seed coat pigmentation (Xie et al., 2019). The analysis of a seed coat color QTL that overlaps with the known *I* locus showed that the W05 reference genome possesses the same inverted repeat of the *CHS* gene cluster as the domesticated soybean reference genome, *G. max* (*Williams* 82). This indicates that additional factors also played a role in causing the seed color changes during domestication. A comparative genomic analysis of W05 against two domesticated soybeans (Wm82 and ZH13) revealed the generation of a small interfering RNA (siRNA) from a large structural rearrangement next to the *CHS* gene cluster in Wm82 and ZH13. Through experimental validation, a subtilisin promoter was shown to drive the expression of a chimeric transcript that reads through a subtilisin gene fragment and an anti-*CHS1* gene region, resulting in PTGS and inhibits the expression of *CHS* genes.

Flavonoids also contribute to floral pigmentation (Tanaka, 2006; Tanaka et al., 2008). Domesticated cowpea (*Vigna unguiculata* L. Walp) shows phenotypic variation compared to its wild relatives. Among the domestication traits, a wide range of floral and seed coat colors can be found in the cultivated cowpea. The wild variety shows purple flowers and dark seed pigmentation. Purple flowers are the results of diacylated delphinidin-based anthocyanins (Tanaka et al., 2008). A QTL analysis of the determinants of floral color in cowpea was performed in a biparental mapping population (wild × cultivated crosses; Lo et al., 2018). A single major QTL for floral color, CFcol7, was mapped in a 64-cM region on chromosome Vu07 containing 254 annotated genes, among which a transcription factor, *Vigun07g110700*, was identified as a homolog of *Arabidopsis AT4G09820.1* and *Medicago truncatula* (*Mt*) *TT8*, involved in the regulation of flavonoid biosynthesis (Nesi et al., 2000; Li et al., 2016). In soybean, one QTL for floral pigmentation was identified on linkage group G (Josie et al., 2007).

#### Defense-Related Traits

**Figure 2:**
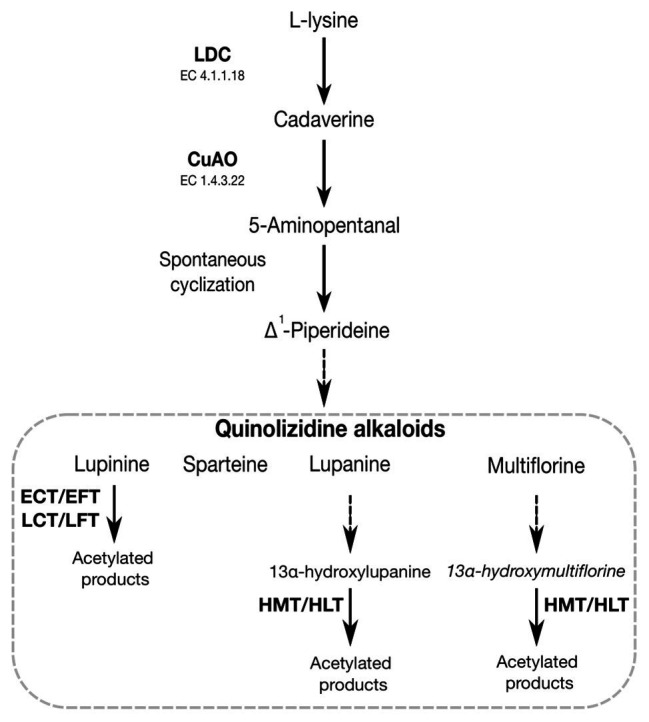
Schematic representation of the pathway leading to the synthesis of quinolizidine alkaloid compounds. Enzymes involved in the pathway are indicated in bold: lysine decarboxylase (LDC), copper amine oxidase (CuAO), (+)-epilupinine *O*-coumaroyltransferase (ECT), (+)-epilupinine feruloyltransferase (EFT), (−)-lupinine *O*-coumaroyltransferase (LCT), (−)-lupinine feruloyltransferase (LFT), (−)-13α-hydroxymultiflorine transferase (HMT), and (+)-13α-hydroxylupanine *O*-tigloyltransferas (HLT).

Toxic secondary metabolites in legumes confer resistance against pathogens and herbivores. However, the accumulation of these compounds in legume crops is not desirable for human consumption or as animal feed since they impart a bitter taste and could present acute toxicity if ingested in sufficient quantities (Daverio et al., 2014). Alkaloids are some of the main secondary metabolites produced and stored by legumes with characteristic toxicity (Wink, 2013). Examples are quinolizidine alkaloids (QAs) produced by the genera *Lupinus*, *Baptisia*, *Thermopsis*, *Genista*, *Cytisus*, *Echinosophora*, and *Sophora* (Ohmiya et al., 1995). The breeding of low-alkaloid (sweet) varieties changed the agronomic roles of lupins from green manure and forage crops to grain legumes with high protein and fiber contents and health benefits (Sweetingham and Kingwell, 2008; Arnoldi et al., 2015). Four species within the genus *Lupinus* have been domesticated and are important legume crops: *Lupinus angustifolius* (narrow-leafed lupin or blue lupin), *L. albus* (white lupin), *Lupinus luteus* (yellow lupin), and *Lupinus mutabilis* (Andean lupin) (Gustafsson and Gadd, 1965; Reinhard et al., 2006). QAs produced by lupins include lupanine, angustifoline, lupinine, sparteine, multiflorine, and aphylline (Frick et al., 2017). The use of lupins for food purposes depends on their QA levels and each species has a characteristic alkaloid composition. Domestication has reduced the amount of alkaloids in lupins, but it has also increased their susceptibility to several aphid species (Philippi et al., 2015). QAs are derived from the decarboxylation of L-lysine (Lys) by a lysine decarboxylase (LDC, EC 4.1.1.18) to form cadaverine (Bunsupa et al., 2012a,b), which is then converted to 5-aminopentanal *via* oxidative deamination by a copper amine oxidase (CuAO, EC 1.4.3.22). 5-aminopentanal is spontaneously cyclized to a Δ^1^ piperideine Schiff base formation (Leistner and Spenser, 1973; Gupta et al., 1979; Golebiewski and Spenser, 1988; Bunsupa et al., 2012b), which then undergoes further modifications (e.g., oxygenation, dehydrogenation, hydroxylation, or esterification) to produce a range of Lys-derived alkaloids, including lupinine, sparteine, lupanine, and multiflorane (Ohmiya et al., 1995; Bunsupa et al., 2012b; Frick et al., 2017). QAs are stored in the form of QA esters. In lupins, QA esters are converted from lupinine/epilupinine and 13α-tigloyloxymultiflorine/13α-tigloyloxylupanine by two types of acetyltransferases (ATs): (+)-epilupinine/(−)-lupinine O-coumaroyl/feruloyltransferase (ECT/EFT-LCT/LFT) and (−)-13α-hydroxymultiflorine/(+)-13α-hydroxylupanine O-tigloyltransferase (HMT/HLT; EC 2.3.1.93), respectively (Saito et al., 1992; Okada et al., 2005; Bunsupa et al., 2012a). The biosynthesis pathway of QAs is illustrated in Figure 2. An HMT/HLT-type acetyltransferase was isolated and characterized at the molecular level in *L. albus* (Okada et al., 2005) while an acyltransferase-like gene, *Lupinus angustifolius acyltransferase* (*LaAT*), has been proposed to be involved in the QA biosynthetic pathway (Bunsupa et al., 2011).

Domestication-related genetic modifications resulting in low-alkaloid phenotypes are generally results of naturally occurring (spontaneous) mutations (Gustafsson and Gadd, 1965). The domestication of lupins led to the active selection by farmers/breeders for sweet varieties which were low in alkaloids. In the late 1920s, the first low-alkaloid lines were obtained from wild germplasms of *L. luteus* and *L. angustifolius* (von Sengbusch, 1942). Subsequently, sweet types were also obtained for *L. albus* and *L. mutabilis* in the 1930s (Taylor et al., 2020). Several recessive low-alkaloid mutations have been discovered in *L. angustifolius*: *iucundus* (*iuc*), *esculentus* (*es*), *depressus* (*depr*), and *tantalus* (Swiecicki and Swiecicki, 1995; Kurlovich, 2002; Taylor et al., 2020), among which, the *iucundus* locus is the most prevalent allele in cultivars (Taylor et al., 2020). Molecular mapping efforts have allowed researchers to map the *iucundus* locus to a 746-kb region on chromosome NLL-07 (Nelson et al., 2006, 2010; Hane et al., 2017). The reference *L. angustifolius* genome also facilitated the identification of markers linked to *iucundus* that are suitable for marker-assisted selection (MAS). Specifically, an allele marker, IucLi, has been identified for the *iucundus* locus, and could be used for MAS in wild × domesticated crosses in lupin breeding programs (Li et al., 2011). Recently, 12 candidate genes for the alkaloid locus *iucundus* and the major QTLs associated with total QA contents were identified using a transcriptomic approach (Kroc et al., 2019b). The most promising candidate, *RAP2-7*, encodes an ethylene-responsive transcription factor (ERF) that co-segregated with the *iucundus* locus and is likely to be involved in the regulation of QA biosynthesis in *L. angustifolius* (Kroc et al., 2019a). Other candidate genes include a *4-hydroxy-tetrahydrodipicolinate synthase* (*DHDPS*) involved in Lys biosynthesis as well as genes involved in plant secondary metabolism (Kroc et al., 2019b).

Recessive low-alkaloid mutations in *L. albus* have also been identified: *pauper*, *mitis*, *reductus*, *exiguus*, and *nutricius* (Hackbarth, 1957; Troll, 1958; Porsche, 1964). As in the case of *L. angustifolius*, one locus, *pauper*, is the most studied in *L. albus* (Rychel and Książkiewicz, 2019). The Kiev mutant × P27174 recombinant inbred lines (RILs) population was used for the first genetic map of *L. albus* where the *pauper* locus was located on linkage group 11 (Phan et al., 2007). Recently, a high-resolution map was developed to provide a high-resolution QTL assay of the agronomic traits of *L. albus* (Michał et al., 2017). The *pauper* locus was localized in the linkage group ALB18 (Michał et al., 2017). The *Lup021586* gene was identified in the region and showed 100% nucleotide identity to *LaAT*, the acyltransferase gene previously identified in *L. angustifolius* (Bunsupa et al., 2011). *LAGI01_35805*, an *L. albus* homolog of *LaAT* that is highly similar to *L. angustifolius Lup021586* gene, has been proposed as a molecular marker for the *pauper* locus (Rychel and Książkiewicz, 2019). Meanwhile, four low-alkaloid alleles have been identified in *L. luteus*, including *dulcis*, *amoenus*, *liber*, and *v* (von Sengbusch, 1942; Gustafsson and Gadd, 1965). However, there is limited information on the genetic basis for the low-alkaloid trait in this species. Efforts to improve the genomic resources of *L. luteus* are underway. The first genetic map for *L. luteus* has been recently released (Iqbal et al., 2019). A high-quality reference genome will help to implement MAS and identify loci responsible for the low-alkaloid content in *L. luteus.*

On the other hand, phytoalexins are a class of secondary metabolites with antimicrobial activities that are synthesized *de novo* after biotic and abiotic stresses (Walton, 1997). Phytoalexin biosynthesis can be induced by pathogens or a type of stress-mimicking compounds called elicitors (Angelova et al., 2006), and are produced by a range of crops including those in the *Fabaceae* family (Ahuja et al., 2012). Phytoalexins produced by the family *Leguminosae* comprise a variety of chemical compounds, including flavonoid phytoalexins derived from the shikimic acid pathway. In species such as soybean, prenylated pterocarpans, i.e., glyceollins, are synthesized in response to fungal pathogens such as *Phytophthora sojae* and *Macrophomina phaseolina* (Lygin et al., 2013). Soybean produces six forms of the isoflavonoid phytoalexin, glyceollin, where glyceollin I, glyceollin II, and glyceollin III are the predominant isomers (Banks and Dewick, 1983), derived from the addition of a dimethylallyl chain to (6aS,11aS)-3,9,6a-trihydroxypterocarpan (glycinol) at either C-4 or C-2 by prenyltransferases (PTs). Two isoflavonoid PTs have been identified in soybean: 4-dimethylallyltransferase (G4DT) and glycinol 2-dimethylallyltransferase (G2DT; Akashi et al., 2009; Yoneyama et al., 2016). Molecular characterization of *PT* genes revealed that *G4DT* and *G2DT* are paralogs resulting from a whole-genome duplication (Yoneyama et al., 2016). A genome-wide analysis of *PT* genes in *G. max* Wm82 identified 77 PT-encoding genes with 11 putative isoflavonoid-specific PTs (Sukumaran et al., 2018). One of the candidate genes, *GmPT01* (*G2DT-2*) was induced by *P. sojae* infection and AgNO_3,_ which mimics pathogen attack and lies in the QTL linked to *P. sojae* resistance. It was suggested that *GmPT01* is one of the genes involved in the partial resistance and could be used in breeding for increased fungal resistance. Other genes related to *P. sojae* resistance include a CHS gene, *GmCHR2A*, located near a QTL linked to *P. sojae* resistance (Sepiol et al., 2017). Additionally, studies have shown that resistant and susceptible genotypes differ in their timing of activating glyceollin biosynthesis (Yoshikawa et al., 1978; Hahn et al., 1985). A rapid activation of the biosynthetic pathway allows a high level of accumulation of these low-molecular weight compounds and confers resistance to pathogens. Soybean genotypes encoding the *P. sojae* resistance gene, *Rps1k*, have shown a rapid activation of glyceollin biosynthesis and higher resistance to the pathogen (Yoshikawa et al., 1978; Hahn et al., 1985). Recently, a member of the NAC (NAM/ATAF1/2/CUC2)-family of transcription factor (TF) genes, *GmNAC42-1*, was identified using comparative transcriptomics (Jahan et al., 2020). GmNAC42-1 binds the promoter of *G4DT* and plays a role in the accumulation of glyceollin I. However, additional TFs are expected to participate in the regulation of glyceollin biosynthesis.

**Figure 3:**
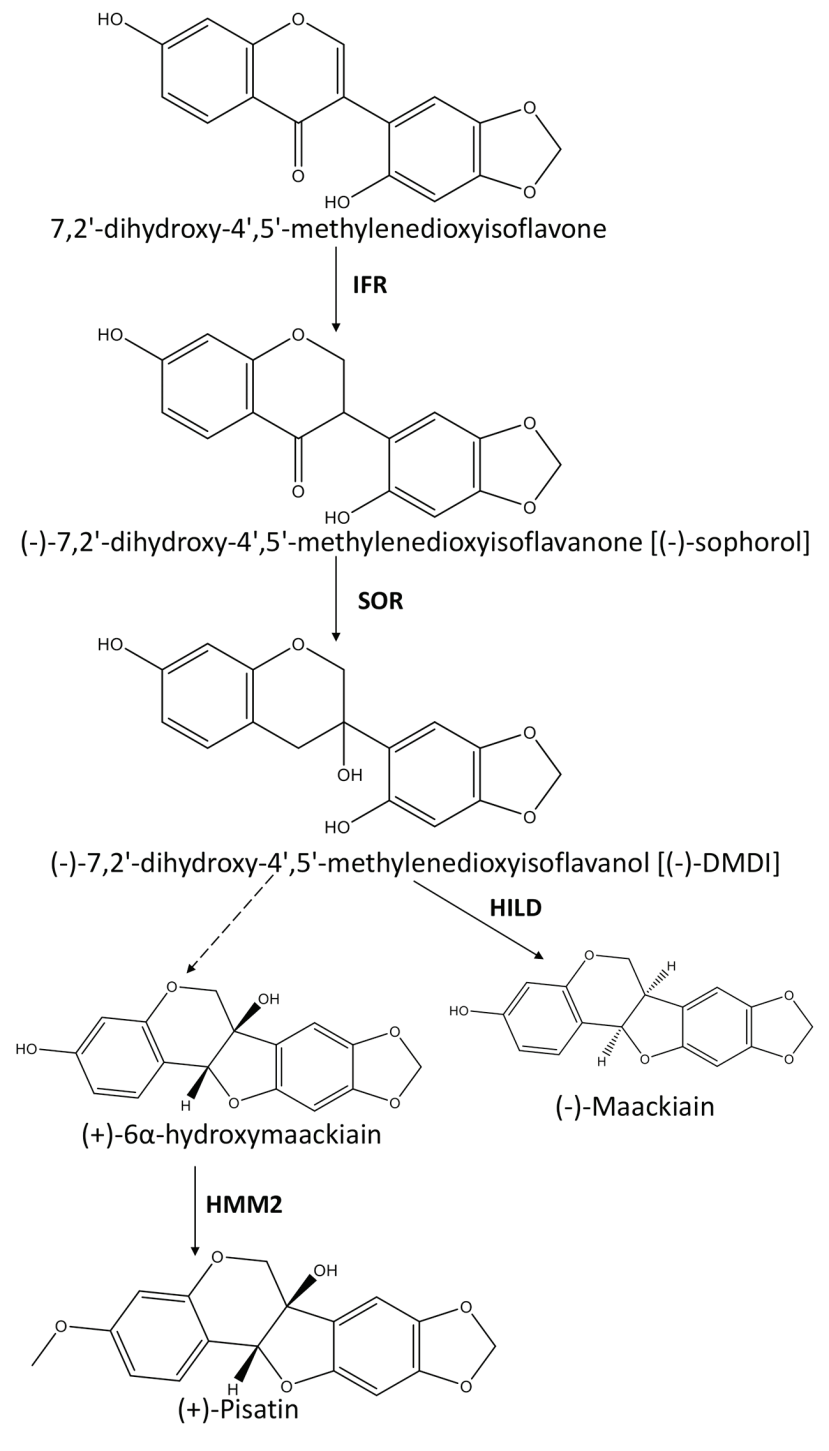
Schematic representation of the pathway leading to the synthesis of (+)-pisatin and (−)-maackiain. Enzymes involved in the pathway are indicated in bold: isoflavone reductase (IFR), sophorol reductase (SOR), (+)-6α-hydroxymaackiain 3-*O*-methyltransferase (HMM2), and hydroxisoflavanol dehydratase (HILD). The steps to convert (−)-7,2'-dihydroxy-4',5'-methylenedioxyisoflavanol (DMDI) to (+)-6α-hydroxymaackiain are unknown (dotted arrow).

The phytoalexins in pea (*P. sativum*) are pisatin and maackiain (Ahuja et al., 2012). Pisatin, a 6α-hydroxyl-pterocarpan phytoalexin, is the major phytoalexin in pea produced in response to fungal infections by *Nectria haematococca*, *Botrytis cinerea*, and *Mycosphaerella pinodes* (Van den Heuvel and Glazener, 1975; Shiraishi et al., 1978; Etebu and Osborn, 2010). Its biosynthetic pathway has been partially characterized (Paiva et al., 1994; DiCenzo and Vanetten, 2006; Kaimoyo and VanEtten, 2008; Celoy and VanEtten, 2014). Pisatin and maackiain are synthetized *via* two chiral intermediates, (−)-7,2'-dihydroxy-4',5'-methylenedioxyisoflavanone [(−)-sophorol] and (−)-7,2'-dihydroxy-4',5'-methylenedioxyisoflavanol [(−)-DMDI; Preisig et al., 1989; Akashi et al., 2006; DiCenzo and Vanetten, 2006]. Sophorol reductase (SOR) is responsible for the production of sophorol, and it can be inactivated by RNA-mediated genetic interference (RNAi) which inhibits the production of pisatin in transgenic pea hairy roots (Kaimoyo and VanEtten, 2008). The pathway diverges for pisatin production after (−)-DMDI formation. In the last step of the pathway, the methylation of (+)-6α-hydroxymaackiain (6α-HMK) at the C-3 position by 6α-hydroxymaackiain methyltransferase (HMM2) results in the formation of (+)-pisatin. (−)-DMDI is converted to (−)-maackiain by hydroxisoflavanol dehydratase (HILD). The biosynthesis pathway of (+)-pisatin and (−)-maackiain is illustrated in Figure 3. *M. pinodes* causes ascochyta blight, the most important foliar disease of field pea, which responds by accumulating pisatin (Shiraishi et al., 1978). Efforts have been made to elucidate the QTLs associated with the disease resistance and to facilitate the introgression of resistance into pea cultivars (Wroth, 1999; Timmerman-Vaughan et al., 2004; Prioul-Gervais et al., 2007; Fondevilla et al., 2011). However, only moderate resistance has been reported with such efforts in pea cultivars (Kraft et al., 1998). Wild relatives present a high phytoalexin diversity (Lindig-Cisneros et al., 2002). The genetic controls for the resistance to *M. pinodes* were studied in a wild *P. sativum* subsp. *syriacum* accession P665 (Fondevilla et al., 2008). Six QTLs associated with *M. pinodes* resistance were detected in linkage groups II, III, IV, and V (Timmerman-Vaughan et al., 2004; Prioul-Gervais et al., 2007). Quantitative trait loci *MpV.1* and *MpII.1* were specific for seedlings under growth chamber conditions and *MpIII.3* and *MpIV.1* for adult plant resistance in field conditions (Timmerman-Vaughan et al., 2004; Prioul-Gervais et al., 2007). In contrast, QTLs *MpIII.1* and *MpIII.2* were detected both for seedling and field resistance (Timmerman-Vaughan et al., 2004; Prioul-Gervais et al., 2007). *MpIII.2* overlaps with a QTL previously reported to be related to the resistance against ascochyta blight complex *Asc3.1* (Timmerman-Vaughan et al., 2004; Prioul-Gervais et al., 2007). A resistance-gene analog (*RGA1.1*) was identified in the vicinity of this QTL using *P. sativum* populations (Timmerman-Vaughan et al., 2004; Prioul-Gervais et al., 2007). QTLs associated with partial resistance to the root rot-causing *A. euteiches* have also been identified in pea, and would be useful for improving and facilitating the existing recurrent selection-based breeding program (Kraft, 1988; Lewis and Gritton, 1992). Pilet-Nayel et al. (2002) crossed susceptible lines with partially resistant ones in order to map the QTLs associated with resistance against *A. euteiches*. The genetic map revealed seven such genomic regions and *Aph1* located on the linkage group IVb was the most promising. Other minor QTLs were also identified in the 13 linkage groups obtained in the genetic mapping (Pilet-Nayel et al., 2002). Meta-analyses of four RILs of pea revealed 27 meta-Aphanomyces resistance QTLs, including 11 with high consistency across populations, locations, years, and isolates (Hamon et al., 2013). Seven highly consistent genomic regions were identified with the potential for use in MAS for pea improvement. Resistance QTLs located in these seven regions were further validated (Lavaud et al., 2015). Backcross-assisted selection programs were used to generate near-isogenic lines (NILs) carrying the resistance alleles of individual or combined resistance QTLs. The effects of two major QTLs, *Ae-Ps4.5*, and *Ae-Ps7.6*, were validated. The NILs carrying the resistance alleles of these two QTLs showed the highest resistance to *A. euteiches* strains. Several minor-effect QTLs were also validated, including *Ae-Ps2.2* and *Ae-Ps5.1*. Genome-wide analyses further validated most of these resistance QTLs and detected additional novel resistance loci (Desgroux et al., 2016). Putative candidate genes in these loci were related to biotic stress responses.

### Transporters

ATP-binding cassette transporters and multidrug and toxic compound extrusion transporters play important roles in the secretion and accumulation of secondary metabolites (Yazaki, 2005; Ku et al., 2020). These transporters are associated with microbe interaction and nutrient accumulation of legumes (Sugiyama et al., 2007; Zhang et al., 2010; Fondevilla et al., 2011; Li et al., 2016).

#### ABC Transporter

In soybean (*G. max*), an ABC transporter was reported to be involved in the root secretion of genistein, which is an important signaling molecule for mediating the symbiosis with rhizobia (Sugiyama et al., 2007). In *M. truncatula*, two half-ABC transporters, STR and STR2, are essential for arbuscule development in arbuscular mycorrhizal symbiosis (Zhang et al., 2010). The expression of the *STR* and *STR2* genes was induced in cortical cells containing arbuscules (Zhang et al., 2010). STR and STR2 dimerize to form a transporter, which is located in the peri-arbuscular membrane and is important for the arbuscule development and therefore the symbiosis (Zhang et al., 2010). The *str* mutant and *STR2*-silenced transgenic roots exhibited stunted arbuscules after inoculating with *Glomus versiforme* (Zhang et al., 2010). In pea, using microarray technology, an ABC transporter was found to have a higher expression in *P. sativum* ssp. *syriacum* accession P665, which is resistant to *Mycosphaerella pinodes*, than the sensitive accession Messire (Fondevilla et al., 2011).

#### MATE Transporter

Seeds of wild soybeans (*G. soja*) generally have higher antioxidant contents than cultivated soybeans (*G. max*; Li et al., 2016). Statistical analysis showed the high correlation among the levels of seed total antioxidants, phenolics, and flavonoids (Li et al., 2016). Using RILs resulted from the cross between the wild soybean W05 (*G. soja*) and the cultivated soybean C08 (*G. max*), QTLs regulating the contents of antioxidants, phenolics, and flavonoids in soybean seeds were identified, which share a common genomic region (Li et al., 2016). In the target genomic region, three genes, *GmMATE1*, *GmMATE2*, and *GmMATE4*, were predicted to encode MATE transporters (Li et al., 2016). These *MATE* genes are possible candidates for investigating the basis behind the different seed antioxidant contents between wild soybeans (*G. soja*) and cultivated soybeans (*G. max*).

## Molecular Breeding and Secondary Metabolite Content

As covered in this review, legumes produce a diverse array of secondary metabolites including a large subset of compounds with biopharmaceutical/nutraceutical properties. The production of these phytochemicals can be increase through crop improvement using classical breeding to genetic approaches (Jacob et al., 2016). Legumes with increased health-beneficial secondary metabolites are potential raw materials for producing pharmaceutical products.

The genetic variability of legume species is fundamental to identify parental lines to be used in breeding programs and exploit legume secondary metabolites. Modern targeted breeding programs use tools, such as quantitative trait loci, marked-assisted selection, and genomics applications (Collard and Mackill, 2008; Jacob et al., 2016). DNA-based molecular markers are used to characterize genomic regions (insertions, deletions, mutations) controlling a particular trait or gene to differentiate individuals for germplasm identification and characterization (Nadeem et al., 2018). Molecular markers provide breeders with a valuable resource to accelerate selection programs and mark complex traits, which are influenced by environmental factors or not observable at early stages of plant development. Flavonoids have pharmacological effects, such as antioxidants for human nutrition or anti-inflammatory effects among others. Also, nutritional value of legumes can be enhanced by increasing flavonoid content though breeding selection (D’Amelia et al., 2018). In this case, molecular markers have been used to study genetic variability in legumes to obtain varieties with high total flavonoid content. Genetic heritability of flavonoids is high and germplasms with different flavonoid content can lead to the identification of potential markers to use in breeding (Caseys et al., 2015). Flavonoid content was determined in 57 peanut accessions to evaluate the association between molecular markers and flavonoid content (Hou et al., 2017). Four expressed sequence tag-simple sequence repeat (EST-SSRs) markers were identified related to high flavonoid content in Chinese peanut germplasm. Functions of these markers were analyzed and related to outer membrane protein porin, heat-shock transcription factor, and lectins (Hou et al., 2017). Further studies are required to confirm the functions of these ESTs in flavonoid synthesis in peanuts. In soybean, three novel alleles were identified associated to flavonoid hydroxylase genes, *F3'H* and *F3'5'H*, related to pigmentation traits (Guo and Qiu, 2013). These molecular markers were identified using a set of gene-tagged markers based on the sequence variation of *GmF3'H* and *GmF3'5'H* in different soybean accessions, including cultivars, landraces, and wild soybeans (Guo and Qiu, 2013). Domestication process does not appear to erode diversity since four *GmF3'H* alleles were identified among cultivated soybeans, while *G. soja* contained only the *GmF3'H* allele. In the case of *GmF3'5'H*, 92.2% of wild soybean contained the *GmF3'5'H-a* allele, while three *GmF3'5'H* alleles occurred among cultivated soybeans (Guo and Qiu, 2013). In white clover (*Trifolium repens*), diversity array technology (DArT) and microsatellite markers were used to discover marker-trait associations for flavonoid accumulation and biomass (Ballizany et al., 2016). Significant associations to concentrations of flavonols quercetin, kaempferol, and Quercetin:Kaempferol ratio were found to markers on linkage group 1–2. Additionally, the study revealed deleterious alleles in an elite cultivar indicating that genetic variability from wild germplasm could be used for white clover improvement (Ballizany et al., 2016).

## Engineering Secondary Metabolite Contents in Legumes

In addition to breeding programs to improve domesticated varieties and broaden the gene pool of cultivars, secondary metabolite contents can also be modified through plant metabolic engineering (DellaPenna, 2001). The identification of genes involved in the biosynthesis pathways of diverse secondary metabolites has helped to drive strategies to optimize the production of target compounds. Increased production of target metabolites can be achieved by altering the primary or secondary metabolism of an organism, for example, through the overexpression of genes in biosynthetic pathways or by knocking out gene expressions and hence the enzymatic activities of competing pathways. In soybean, the manipulation of the (iso)flavonoid pathway and its effect on the resistance to *P. sojae* has been studied (Cheng et al., 2015; Chen et al., 2018; Zhou et al., 2018). *GmIFR*, encoding an isoflavone reductase (IFR), was identified and overexpressed in soybean (Cheng et al., 2015). IFR catalyzes an intermediate step in the biosynthesis of glyceollins (Graham et al., 1990) and its constitutive expression in transgenic soybean plants enhances the resistance to *P. sojae*, along with higher glyceollin contents. Similar effects on pathogen resistance were obtained by the overexpression of *coenzyme A ligase* (*GmPI4L*) in transgenic soybean plants (Chen et al., 2018). Further attempts to elucidate enzymes/genes responsible for the resistance to pathogens include the overexpression of a chalcone isomerase, *GmCHI1A*, in soybean hairy roots, which enhanced daidzein accumulation and resistance to *P. sojae* strain P6497R compared to the control (Zhou et al., 2018). In alfalfa, nutritional value was increased by engineering genistein glucoside production (Deavours and Dixon, 2005). A transgenic alfalfa was developed by constitutively expressing an isoflavone synthase, *MtIFS1*, from *M. truncatula*. However, in the *MtIFS1*-expressing transgenic alfalfa, isoflavonoid production and accumulation was tissue-specific and affected by environmental factors such as UV-B and the disease-causing pathogen, *Phoma medicaginis* (Deavours and Dixon, 2005). RNAi-mediated gene silencing of isoflavone reductase, SOR, and hydroxymaackiain-3-*O*-methyltransferase in pea (*P. sativum*) allowed the identification of DMDI, an intermediary in the production of pisatin and maackiain (Kaimoyo and VanEtten, 2008). Other viable strategies of engineering secondary metabolite pathways include biosynthesis in microorganisms and modulation of gene expressions through manipulating the expressions of transcription factors (Du et al., 2010). Recently, genome-scale models have been used to represent the metabolic capabilities of legumes, including alfalfa and soybean (Pfau et al., 2018; Moreira et al., 2019). This approach allows the integration of different kinds of omics data to get new insights into plant-microbe interactions (diCenzo et al., 2016; Pfau et al., 2018; Contador et al., 2020). Models of plant metabolic pathways could also be used in the design of optimal-use biosynthesis pathways of secondary metabolites.

## Conclusion

Domestication generally results in the reduction in secondary metabolites, which are often related to the bitter taste of seeds and the resistance of plants to biotic stresses. The phenomenon is consistent with the reported decrease in crop biodiversity due to domestication (Food and Agricultural Organization of the United Nations, 2010). Having numerous health-beneficial secondary metabolites, legumes have the great potential to be employed as the sources of bioactive compounds for pharmaceutical use. On the other hand, besides abiotic stresses, the changing climate may also bring forth unpredictable biotic stresses such as insect infestations. From these perspectives, it is important to retain the biodiversity of legumes in order to maintain a healthy gene pool to produce new cultivars that can respond to future changes in their environments. Understanding the genes that govern the beneficial secondary metabolite compositions in legumes will facilitate the use of wild legumes in breeding programs or metabolic engineering to promote crop diversity, as well as to produce legumes with favorable secondary metabolite profiles.

## Author Contributions

H-ML planned and coordinated the writing. Y-SK put together the first complete draft and prepared the table. CC drew Figures 1, 2. M-SN prepared the table and drew Figure 3. All authors contributed to the search of literature and writings. All authors contributed to the article and approved the submitted version.

## Disclaimer

Any opinions, findings, conclusions or recommendations expressed in this publication do not reflect the views of the Government of the Hong Kong Special Administrative Region or the Innovation and Technology Commission.

### Conflict of Interest

The authors declare that the research was conducted in the absence of any commercial or financial relationships that could be construed as a potential conflict of interest.
